# Antisense oligonucleotides targeting basal forebrain ATXN2 enhances spatial memory and ameliorates sleep deprivation‐induced fear memory impairment in mice

**DOI:** 10.1002/brb3.3013

**Published:** 2023-04-18

**Authors:** Tao Ma, Long Feng, Shi‐Nan Wei, Ying‐Ying Wang, Guan‐Hua Li, Yan Lu, Ying‐Xin Zhang, Yang Chu, Wei Wang, Hao Zhang

**Affiliations:** ^1^ Department of Anesthesiology PLA Rocket Force Characteristic Medical Center Beijing China; ^2^ Department of Anesthesiology PLA General Hospital of Hainan Hospital Hainan China; ^3^ PLA Rocket Force Characteristic Medical Center, Postgraduate Training Base of Jinzhou Medical University Beijing China; ^4^ Department of Anesthesiology Beijing Ditan Hospital, Capital Medical University Beijing China; ^5^ Department of Neurology PLA Rocket Force Characteristic Medical Center Beijing China

**Keywords:** antisense oligonucleotides, ATXN2, memory, sleep deprivation

## Abstract

**Introduction:**

Regulation of brain‐derived neurotrophic factor (BDNF) in the basal forebrain ameliorates sleep deprivation‐induced fear memory impairments in rodents. Antisense oligonucleotides (ASOs) targeting ATXN2 was a potential therapy for spinocerebellar ataxia, whose pathogenic mechanism associates with reduced BDNF expression. We tested the hypothesis that ASO7 targeting ATXN2 could affect BDNF levels in mouse basal forebrain and ameliorate sleep deprivation‐induced fear memory impairments.

**Methods:**

Adult male C57BL/6 mice were used to evaluate the effects of ASO7 targeting ATXN2 microinjected into the bilateral basal forebrain (1 μg, 0.5 μL, each side) on spatial memory, fear memory and sleep deprivation‐induced fear memory impairments. Spatial memory and fear memory were detected by the Morris water maze and step‐down inhibitory avoidance test, respectively. Immunohistochemistry, RT‐PCR, and Western blot were used to evaluate the changes of levels of BDNF, ATXN2, and postsynaptic density 95 (PSD95) protein as well as ATXN2 mRNA. The morphological changes in neurons in the hippocampal CA1 region were detected by HE staining and Nissl staining.

**Results:**

ASO7 targeting ATXN2 microinjected into the basal forebrain could suppress ATXN2 mRNA and protein expression for more than 1 month and enhance spatial memory but not fear memory in mice. BDNF mRNA and protein expression in basal forebrain and hippocampus was increased by ASO7. Moreover, PSD95 expression and synapse formation were increased in the hippocampus. Furthermore, ASO7 microinjected into the basal forebrain increased BDNF and PSD95 protein expression in the basal forebrain of sleep‐deprived mice and counteracted sleep deprivation‐induced fear memory impairments.

**Conclusion:**

ASOs targeting ATXN2 may provide effective interventions for sleep deprivation‐induced cognitive impairments.

## Graphical abstract

Regulation of Brain‐derived neurotrophic factor in the basal forebrain ameliorates sleep deprivation‐ induced fear memory in rodent animals. Antisense oligonucleotides targeting ATXN2 was a potential therapy for spinocerebellar ataxia, whose pathogenic mechanism associates with the BDNF expression reduction. ASOs reducing gene and protein expression of ATXN2 in the basal forebrain increases the expression of BDNF in the basal forebrain and hence regulates BDNF and PSD95 protein expression in the hippocampus, which in turn enhances spatial memory and counteracts the impairment of fear memory caused by sleep deprivation.

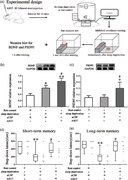

## 1 INTRODUCTION

Sleep plays a crucial role in memory stabilization, but many people suffer from insufficient sleep (Cousins & Fernández, 2019), which is called sleep deprivation. Sleep deprivation greatly affects learning and memory as well as emotion (Krause et al., 2017). The mammalian basal forebrain plays important roles in controlling sleep and wakefulness, and activation of the cholinergic neurons in basal forebrain has been found to enhance arousal, attention, and memory (Xu et al., 2015).

The brain‐derived neurotrophic factor (BDNF) mediates the plasticity‐related changes that associate with memory processing during sleep. Enhancement of BDNF signaling has become a novel therapeutic approach for neurodegenerative and neuropsychiatric disorders (Bawari et al., 2019; Rahmani et al., 2020). Enhancement of BDNF signaling could partly reverse the detrimental effects of sleep deprivation on memory (Looti Bashiyan et al., 2021; Ma et al., 2020). Many delivery methods of exogenous BDNF have been investigated for treatment of neurodegenerative diseases, including viral microbubbles, nanoparticles, ultrasound assisted technology, etc. However, it is still a challenge due to the short half‐life, poor bioavailability, and marginal permeability through the blood‐brain‐barrier (BBB) (Nagahara & Tuszynski, 2011; Wang et al., 2021). Furthermore, the duration of treating chronic neurodegenerative disorder may last for years. There was a great need to explore drugs with long‐lasting promoting effects on BDNF expression.

ATXN2 is one of the few genes that a single gene causes several diseases and/or modifies several disparate neurological disorders (Laffita‐Mesa et al., 2021). Spinocerebellar ataxia type 2 (SCA2) is autosomal dominantly inherited and caused by DNA CAG repeat expansion leading to an increase in the polyglutamine (polyQ) domain in the N‐terminal part of the ATXN2 protein. The clinical spectra of SCA2 include motor dysfunctions and cognitive disturbances in executive function and memory (Gigante et al., 2020). Antisense oligodeoxynucleotides (ASOs) targeting ATXN2 mRNA might be useful for treating SCA2. One study screened 152 ASOs targeting ATXN2 mRNA and found ASO7 could downregulate ATXN2 mRNA and protein for more than 12 weeks, resulting in delayed onset of disease phenotypes in SCA2 model mice (Scoles et al., 2017). Furthermore, the SCA pathogenic mechanism includes BDNF mRNA expression reduction and positive regulation of BDNF could ameliorate motor deficits and cerebellar pathology of SCA (Hourez et al., 2011; Sheeler et al., 2021; Takahashi et al., 2012). But whether ASOs targeting ATXN2 also increase BDNF expression is currently unknown. In this study, we proposed and tested the hypothesis that ASOs targeting ATXN2 in the basal forebrain could increase BDNF expression, thereby modulating sleep deprivation‐induced memory impairments.

## 2 MATERIALS AND METHODS

### 2.1 Animals and sleep deprivation

Adult male C57BL/6 mice (22 ± 5 g) from Experimental Charles River Laboratories, Beijing, China, were used for this study. Procedures involving animals were conducted in accordance with the Guide for Care and Use of Laboratory Animals. All animals were housed individually under controlled conditions (12 h/12 h light‐dark cycle, lights on at 08:00) in an isolated ventilated chamber maintained at 23 ± 1°C with ad libitum access to food and water. The experiments began when all animals had been acclimated to the environmental conditions for at least 2 weeks. Mice for sleep deprivation were placed inside a sleep deprivation device, which consisted of a cylinder and an inbuilt interfering rod (XR‐XS108, Xinruan Information Technology, Shanghai, China). The mice were allowed to move freely in the cylinder. In order to avoid sleeping, the interfering rod rotates consistently at a constant speed (15 rpm) in the cylinder. The process of sleep deprivation started at 8:00 am and ended at 2:00 pm.

### 2.2 Experimental design

We proposed and verified the hypothesis that ASOs targeting the ATXN2 in the basal forebrain could regulate BDNF expression, thus modulating sleep deprivation‐induced memory impairments. To achieve this goal, this study comprised the following two parts.

**FIGURE 1:**
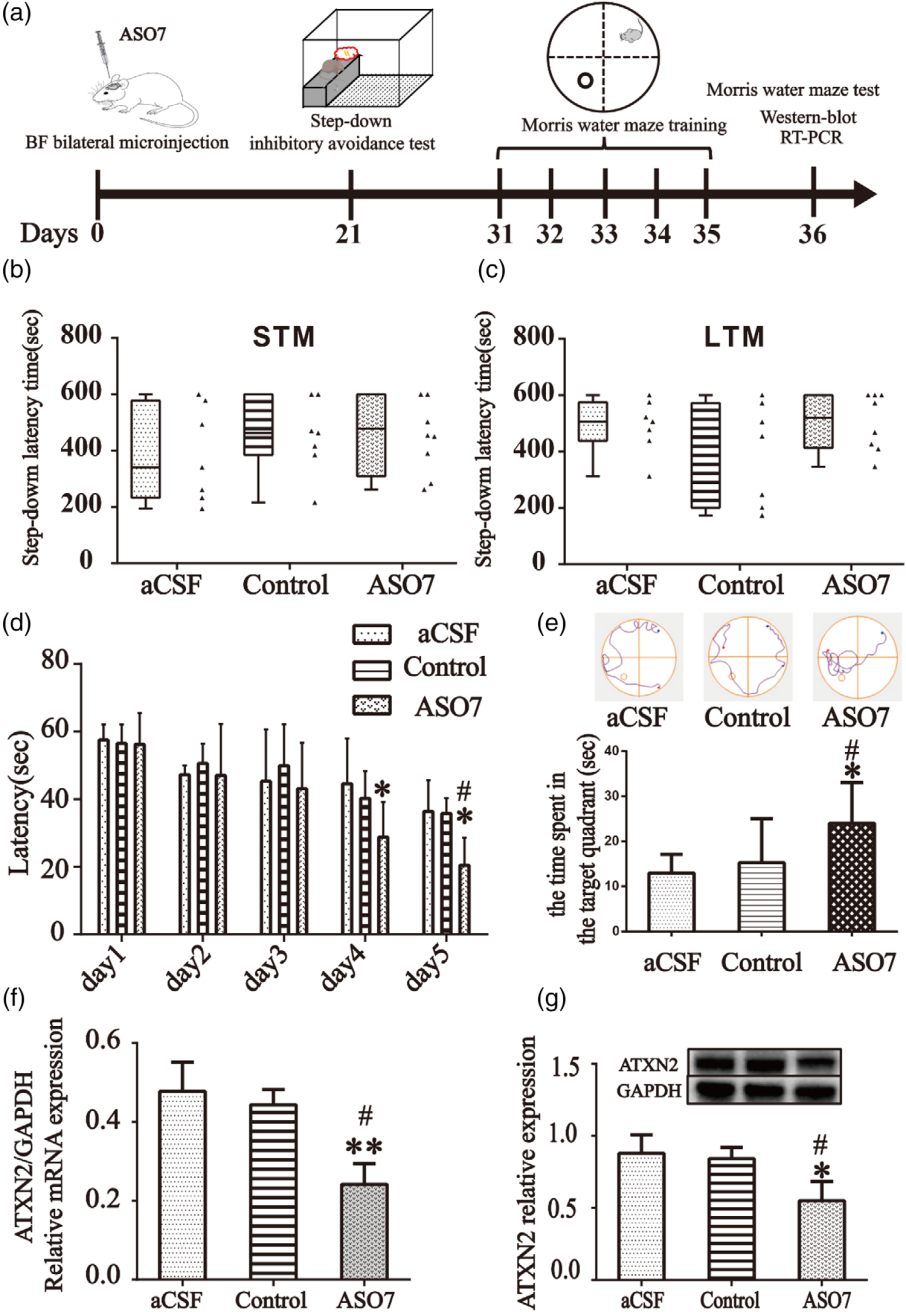
**Microinjection of ASO7 into basal forebrain imposes no effect on fear memory, but enhances spatial learning and memory. (A)** Schematic design. 21 days after ASO7 microinjection, fear memory was evaluated. After 30 days of microinjection, mice received Morris water maze test. RT‐PCR and Western blot were carried out after the water maze test. Box and whiskers plots (min to max) combined with scatter plots showing ATXN2 inhibition in basal forebrain has no effect on **(B)** short‐term and **(C)** long‐term fear memory**. (D)** Latency during the navigation training phase. **(E)** Representative video tracks of probe trial and histograms showing the time spent in the target quadrant. **(F)** Ataxin‐2 mRNA changes after ASO7 treatment. **(G)** Ataxin‐2 protein expression after ASO7 microinjection. Values were presented as means ± standard deviations (SDs). ^*^ *p* < 0.05 compared to the aCSF group. ^#^ *p* < .05 compared to the negative control group.

First, animals were divided into ASO7, ASO7 negative control, and artificial cerebrospinal fluid (aCSF) group depending on the drug microinjected into the basal forebrain (*n* = 10 per group). 21 days after drug microinjection, step‐down inhibitory avoidance test was performed to evaluate fear memory. Spatial learning and memory were evaluated 30 days after drug microinjection using the Morris water maze. After these, the mice were euthanized and decapitated. The brains of mice were coronally sectioned by using the Mouse Brain Slicer Matrix to verify the injection site visually. The data from mice with correct microinjection sites were analyzed (*n* = 8, 7, and 7 for ASO7, ASO7 negative control, and aCSF group, respectively). The basal forebrain and the hippocampus were collected for RT‐PCR and Western blot test to observe mRNA and protein expression (Figure 1A). Then 32 mice were used to examine the long‐term effects of ASO7 on basal forebrain BDNF expression, hippocampal BDNF, and postsynaptic density protein‐95 (PSD‐95) protein expression as well as hippocampal synaptic structural plasticity evaluated by Golgi‐Cox staining. 24 mice with correct tip sites were included for data analysis 30 days after microinjection of ASO7 into the bilateral basal forebrain.

Second, ASO7 was microinjected bilaterally into the basal forebrain to observe whether ATXN2 inhibition reversed sleep deprivation‐induced memory impairments by increasing expression of BDNF and PSD95 in the basal forebrain (Figure 3A). Mice were randomly divided into three groups (*n* = 12 per group) and subjected to rest control or sleep deprivation combined with ASO7 treatment or aCSF treatment. Specifically, 1 h after inhibitory avoidance training, four mice per group were euthanized to collect basal forebrain samples for Western blot examination of BDNF and PSD95 expression. The other mice were subjected to both short‐term memory and long‐term memory tests. After measuring long‐term memory, the correct microinjection sites were confirmed by visual inspection. Forty‐nine mice were sacrificed and 36 mice with correct tip site (12 mice per group) were included for data analyses. The mice with missed cannula placements were 5 in rest control plus aCSF group, 4 in sleep deprivation plus aCSF group and 4 in sleep deprivation plus ASO7 group, respectively.

### 2.3 Basal forebrain microinjection

ASO7 (sequence GTGGGATACAAATTCT AGGC, position 88209) was a custom product from Ribobio, China, which included five 2’‐O‐methoxyethyl (MOE) modified nucleotides at each end of the oligonucleotide, with 10 DNA nucleotides in the center, and were phosphorothioate modified in all positions. Microinjection of ASO7 into bilateral basal forebrain was performed using a 10‐μL Hamilton micro syringe (Hamilton, Switzerland) with the tip located at basal forebrain (relative to the bregma: anterior‐posterior [AP] 0.1 mm, medial‐lateral [ML] ±1.2 mm, and dorsal‐ventral [DV] −5.2 to 5.4 mm) under general anesthesia, as described previously (Xu et al., 2015). The dosage of ASO7 was 1 μg (0.5 μL per side). Equal volume of aCSF (Campos‐Jurado et al., 2019) or ASO7 negative control was used as vehicle control and negative control, respectively. The dosage was based on a previous study (Scoles et al., 2017). The drug microinjection duration lasted for 2 min. After the injection, the syringe was left in place for 5 min to allow drug diffusion.

### 2.4 Morris water maze (MWM) test

MWM testing was conducted 10 days after step‐down inhibitory avoidance task for assessment of spatial learning and memory, which was conducted in a circular tank (diameter, 118 cm; height, 44 cm) filled with white nontoxic paint in a dimly lit room. The water temperature was continuously monitored and kept at 24 ± 1°C. The maze was divided into four equal quadrants and a platform (diameter, 11 cm) was placed 1 cm below the surface of water in the center of one quadrant. Several spatial cues with different geometries were placed on the poolsides to help the mice locate the platform. During the training session, the mice were trained for 5 consecutive days with four trials per day. The trial was completed when the mouse found the platform or when 60 s of test time had expired. Once the mouse failed to find the platform within 60 s, it was guided to the platform and stayed on the platform for 15 s with manual assistance. After that, the mouse was being transferred back to cage and the next mouse was tested. This rotation was repeated till all animals had completed their trials. After 5 days of training, a single 60‐s probe trial was performed on the next day. Swimming velocity and the latency to the platform during training days and the time spent in the target quadrant during the probe trial were recorded using ANY‐maze behavioral tracking software system (Stoelting Co. Wood Dale, IL, USA) and analyzed.

### 2.5 Step‐down inhibitory avoidance test

The step‐down inhibitory avoidance task was performed in a cubic (50 × 50 × 25 cm) chamber. There is one rubber safe platform on the floor with 16 parallel copper bars, which can be connected to an electric stimulator. When the mouse was placed on the platform, it was allowed for free exploration in the chamber for 300 s. Then, the animals were subjected to a training session. In the training session, the mouse received a 0.4 mA scrambled foot shock for 2 s when stepping down. One hour after the training session, the mouse was placed on the safe platform again. Latency to step down (four paws on the grids) was recorded as the level of short‐term memory retention. Long‐term memory was evaluated 24 h after training. The maximal observation time was 600 s.

### 2.6 Tissue extraction

Fresh basal forebrain tissues were harvested as described in a previous study (Ding & Toth, 2006). Briefly, basal forebrain was removed from the coronal slice cut with a coronal brain slicer matrix (RWD, China). The 1‐mm‐thick brain slice was approximately at the level of the optic chiasm (–0.014 mm anterior to and 0.34 mm posterior to bregma). Basal forebrain was removed from the slice based on the visual landmarks of the anterior commissure, third ventricle, striatum, and olfactory tubercle. After completion of the MWM, mice were sacrificed by dislocation and the brains were dissected on ice to obtain the hippocampus for BDNF and PSD95 protein examination. The tissues were immediately frozen with liquid nitrogen and stored at –80°C.

### 2.7 Quantitative real‐time PCR

Total RNA was extracted using the Trizol® kit (Sangon Biotech, China) according to the manufacturer's instructions. Equal amounts of RNA were used to synthesize cDNA using the cDNA Synthesis kit (Takara, Japan). qPCR was performed using the SYBR‐Green Supermix (Sangon Biotech, China). The primers used in the RT‐PCR were as follows: ATXN2, 5’‐AAGATACAGACTCCAGTTATGCACGG‐3’ (forward) and 5’GCTCCAGGTCCTTCTCCTTGTGC‐3’ (reverse). All reactions were performed in triplicate. GAPDH was used as an internal reference.

### 2.8 Immunohistochemistry

The hippocampal tissues were harvested and fixed with 4% paraformaldehyde in PBS to investigate BDNF and PSD95 protein expression after finishing MWM. Then, hippocampal tissues sections (5 μm) from the brain were cut and blocked with Tris‐buffered saline containing 0.1% Tween 20 (TBST) and 5% goat serum for 1 h at room temperature. The slides were incubated overnight at 4°C with a rabbit anti‐BDNF antibody (1:200; ab108319, Abcam, USA) or PSD95 (1:100; ab238135, Abcam, USA). On the following day, sections were washed three times and incubated at room temperature with goat anti‐rabbit IgG H&L (1:5000, ab6717, Abcam, USA). Hippocampal slices from each mouse were analyzed using imaging observation equipment (LEICA DMI8, LEICA, Germany) to observe immunohistochemistry staining. Cases were scored as negative if no or only weak BDNF or PSD95 staining was observed. Three sections were obtained from each mouse (*n* = 4 in each group), and three visual fields were randomly selected and observed in each tissue section. The cells were counted, and the mean rates of positive cells from each mouse were compared between the aCSF group and ASO7 group. All quantitative analyses were performed by an experimenter blinded to the group assignment.

### 2.9 Western blot

Proteins from the basal forebrain or hippocampus were extracted according to the manufacturer's instructions (C500007, Sangon Biotech, China), and then separated by SDS‐polyacrylamide gel electrophoresis. After separation, the proteins were transferred onto polyvinyl difluoride membranes (10600023, GE, USA). After blocking, the membranes were incubated overnight at 4°C with rabbit anti‐BDNF (1:2000; ab108319, Abcam, USA), rabbit anti‐PSD95 (1:2000; ab238135, Abcam, USA), or GAPDH (1:20,000; ab181603, Abcam) antibody. On the following day, membranes were washed then incubated for 1 h at room temperature with Tris‐buffered saline containing 0.1% Tween 20 (TBST) and the horseradish peroxidase (HRP)‐conjugated secondary antibody IgG‐HRP (ab6721, Abcam). Densitometric analysis was performed using the BioRad Western blot detection system. GAPDH was used as the internal control.

### 2.10 Golgi‐Cox staining and dendritic spine counting

Golgi‐Cox staining was carried out for visualization of synaptic structural plasticity in the hippocampus. The Golgi‐Cox staining solution kit (Servicebio, China) was employed according to the manufacturer's instructions. Specifically, the brain tissues were immersed in Golgi‐Cox staining solution for 48 h and then changed to a new staining solution once, followed by a new staining solution every 3 days for a total of 14 days in the dark at room temperature. Next, brain tissues were washed 3 times in distilled water and then submerged in 80% glacial acetic acid overnight, and when the tissues became soft, they were washed in distilled water and placed in 30% sucrose. After that, tissue was cut to 100 μm and attached to gelatin slides to dry overnight in the dark. On the following day, the dried tissue slides were treated with concentrated ammonia for 15 min, washed with distilled water for 1 min, treated with acidic firm film fixing solution for 15 min, washed with distilled water for 3 min, dried, and sealed with glycerol gelatin. A Nikon DS‐U3 was used for the photo capture. Analysis of the dendrites and dendritic spines within the CA1 region of the hippocampus was accomplished using the ImageJ software.

### 2.11 Statistical analyses

Statistical analyses were performed using SPSS 18.0 software (IBM). As the fear memory data did not follow a normal distribution, the data were ranked and the ranks were analyzed them using one‐way repeated measures analysis of variance (ANOVA) with the post hoc Bonferroni's multiple comparisons test as previously reported (Zhuang et al., 2018). Data are presented as the medians and interquartile ranges combined with scatter plots in the Figures. The MWM data were analyzed using two‐way ANOVA with post hoc Bonferroni's pairwise comparison and were presented as means ± standard deviations (SDs). Western blot, immunohistochemistry data, and spine density are presented as means ± SDs. One‐way ANOVA with post hoc Bonferroni's test was used for pairwise comparison. Statistical significance was set at *p* < .05 (two‐sided).

## 3 RESULTS

### 3.1 Microinjection of ASO7 into the basal forebrain imposes no effect on fear memory but enhances spatial learning and memory

No significant difference in short‐term (*F*_2.19_ = 0.678, *p* = .519, Figure 1B) and long‐term fear memory (*F*_2.19_ = 0.811, *p* = .459, Figure 1C) were observed among groups.

Two‐way repeated measures ANOVA revealed a significant group (*F*_2.19_ = 5.341, *p* = .014) and time (*F*_4.19_ = 22.565, *p* = .001) effect on the swimming latency (Figure 1D). Mice receiving ASO injection had shortened mean latency compared with animals receiving vehicle control (*p* = .040) or negative control (*p* = .030). Post hoc analysis showed that there was no significant difference in the latency on day 1 (*F*_2.19_ = 0.068, *p* = .934), day 2 (*F*_2.19_ = 0.286, *p* = .754), or day 3 (*F*_2.19_ = 0.463, *p* = .636) among three groups. On day 4, the latency to reach the platform in the ASO7 showed no significant difference compared with negative control group (*p* = .166) but was shorter compared with aCSF group (*p* = .033). On day 5, the latency in ASO7 group was shorter compared with aCSF group (*p* = .002) and negative control group (*p* = .003). The time spent in the target quadrant in probe trial were significantly longer in ASO7 group than aCSF group (*p* = .022) or AOS7 negative control group (*p* = .040) and there was no significant difference between aCSF group and AOS7 negative control group (*F*_2.19_ = 5.638, *p* = 1.000). The representative video tracks of probe trial were showed in Figure 1E.

qRT‐PCR examination showed a significant decrease in ATXN2 mRNA expression 35 days after bilateral basal forebrain injection of ASO7 (*F*_2, 6_ = 15.02, both *p* < .05, Figure 1F). A significant decrease in ATXN2 protein expression by ASO7 was also observed (*F*_2, 6_ = 19.96, both *p* < .05, Figure 1G).

### 3.2 Microinjection of ASO7 into the basal forebrain increases BDNF protein expression in the basal forebrain and regulates BDNF and PSD95 protein expression and synapse formation in hippocampus

**FIGURE 2:**
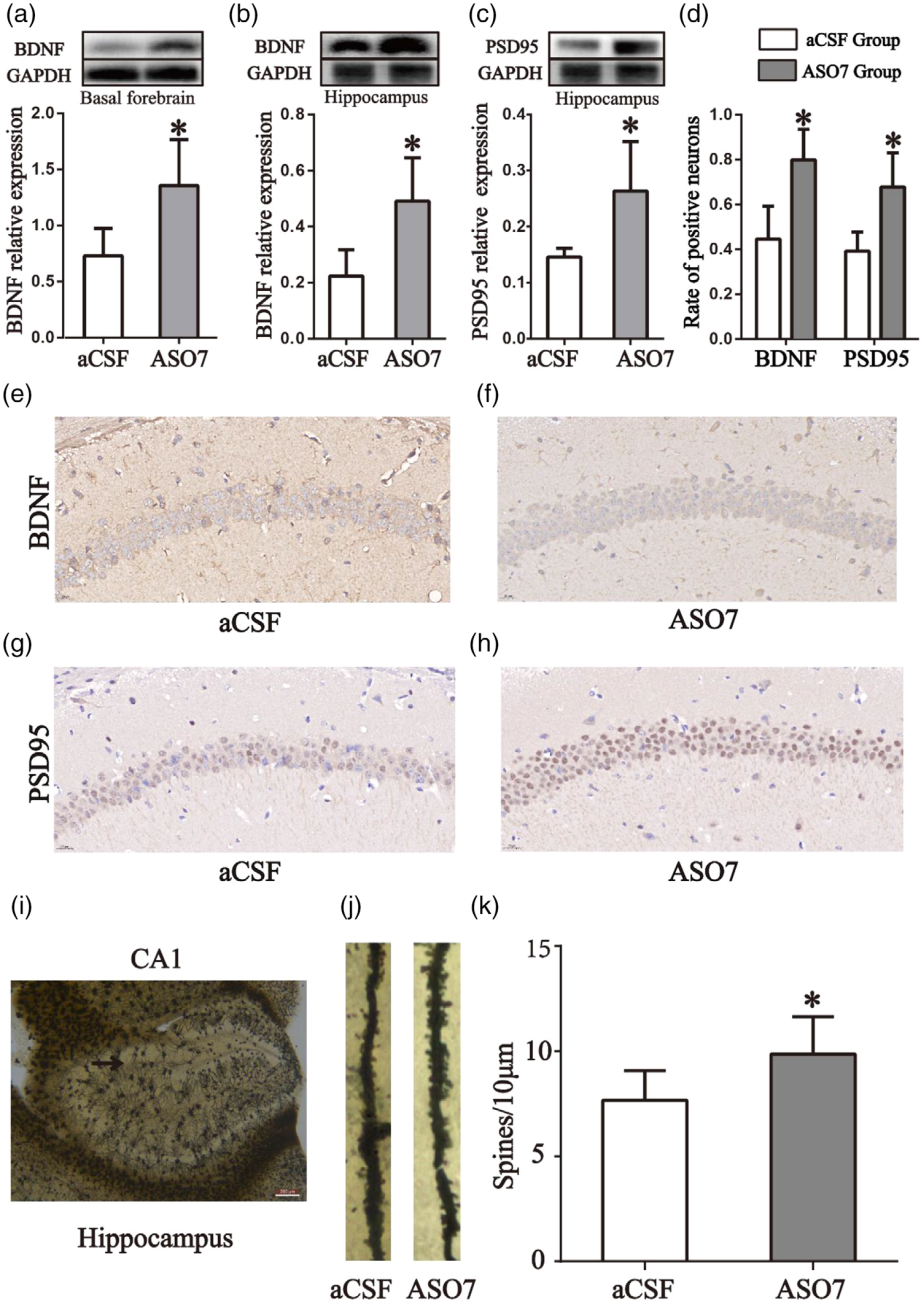
**Microinjection of ASO7 into basal forebrain increases BDNF and PSD95 protein expression and synapse formation in the hippocampus. (A)** Representative Western blot images and histograms showing that ASO7 increased brain‐derived neurotrophic factor (BDNF) protein expression in the basal forebrain. (**B**) Representative Western blot images and histograms showing ASO7 increased BDNF protein expression in the hippocampus. **(C)** Representative Western blot images and histograms showing ASO7 increased PSD95 protein expression in the hippocampus. Histograms (**D**) and representative immunohistochemical pictures (**E–H**) showing ASO7 increased BDNF and PSD95 expression in the hippocampus. **(I–K**) Spine density was quantified in the CA1 region of the dorsal hippocampus from ASO7 and aCSF group mice. (**I**) Representative images of the hippocampal CA1 region analyzed for spine density. (**J**) Representative images of spine density from aCSF and ASO7 group mice. (**K**) ASO7 treatment significantly increased spine density levels of mice compared to aCSF‐treated mice. *p < .05. Scale bar = 200 μm. Data were expressed as means ± SDs. Note: Western blot gel images of subpanels B and C were obtained from the same hippocampus tissues and the same GAPDH gel image was used.

Western blot results showed a significant increase in BDNF protein expression in the basal forebrain after ASO7 microinjection (*t* = 2625, *p* = .0393, Figure 2A). BDNF and PSD95 protein expression in the hippocampus were also increased 35 days after ASO7 microinjection (*t* = 2.954, *p* = .0255; *t* = 2.632, *p* = .0390, Figure 2B and C). Immunohistochemical staining showed that the proportions of BDNF‐positive and PSD95‐positive cells in hippocampus CA1 field were significantly increased in ASO7 group compared with those of the control group (*t* = 3.525, *p* = .0124; *t* = 3.270, *p* = .0170, Figure 2D–H). Compared with aCSF group mice, ASO7 group had significantly larger spine dendritic numbers (*t* = 2.910, *p* = .0102, Figure 2I–K).

### 3.3 ASO7 partly counteracts acute sleep deprivation‐induced fear memory impairments

**FIGURE 3:**
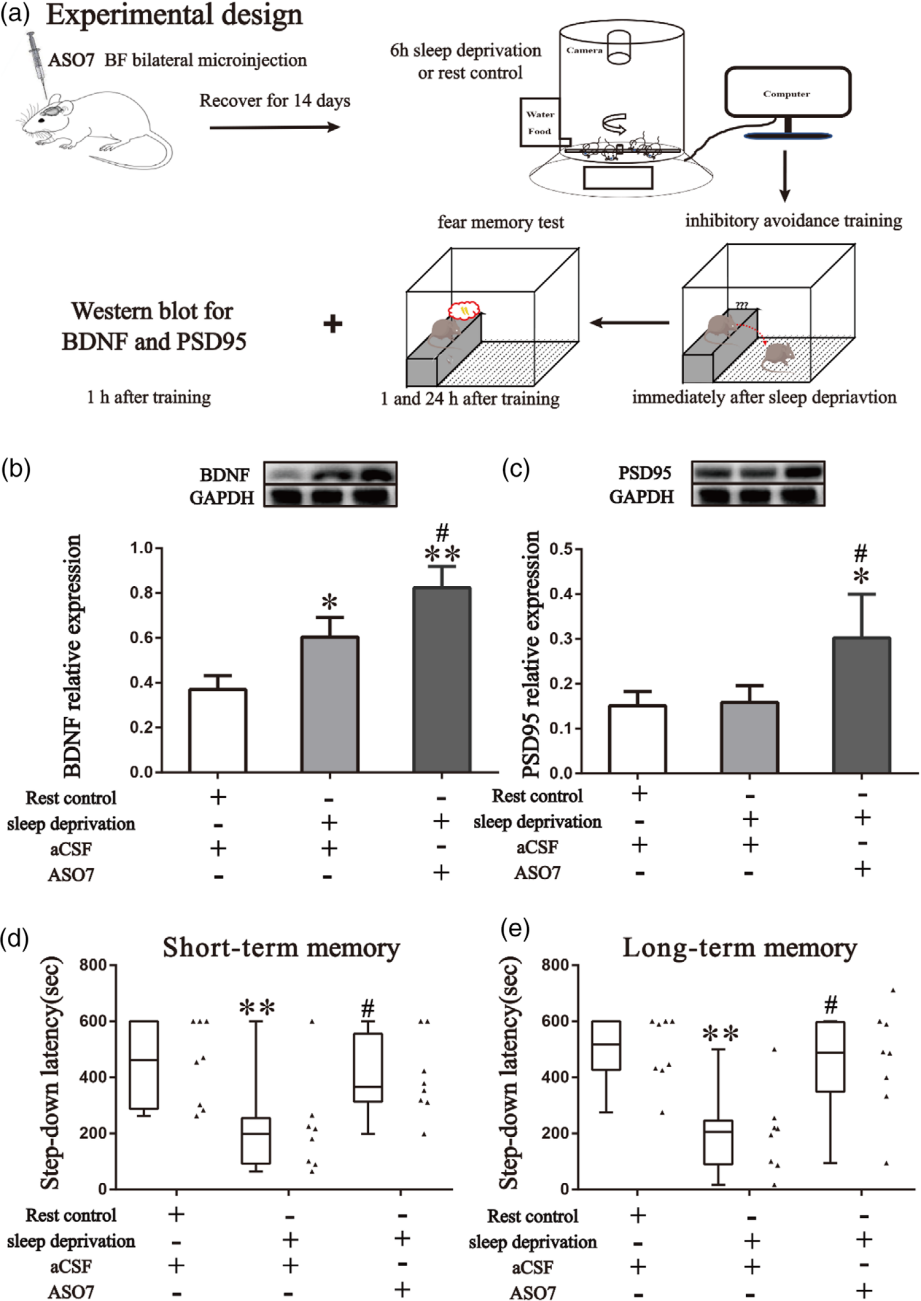
ASO7 increases BDNF and PSD95 protein expression, and partly recues short‐ and long‐term fear memory impairments induced by sleep deprivation. (A) Schematic design. ASO7 (1 μg/0.5 μL/side) or aCSF (0.5 μL/side) were microinjected bilaterally into the basal forebrain of adult male mice. After 14 days, the mice were subjected to 6 h of total sleep deprivation or rest control followed by inhbitory avoidance training. The effects of basal forebrain ATXN2 inhibition on BDNF and PSD95 protein expression and short‐and long‐term memories were then detected. **(B)** Representative Western blot images and histograms showed incresed BDNF expression in the basal forebrain after sleep deprivation, which was enhanced by ASO7. **(C)** Representative Western blot images and histograms showed no change in PSD95 protein expression in basal forebrain after sleep deprivation, but increased PSD95 protein expression was detected in sleep‐deprived mice with ASO7 treatment. **(D, E)** Box and whiskers plots (min to max) combined with scatter plots showing the partial rescue of sleep deprivation induced (F) short**‐term and** (G) long‐term term fear memory impairments by ASO7. *p < .05; **p < .01 compared to the rest control group. ^#^ *p* < .05 compared to the sleep deprivation group. Data were expressed as means ± SDs for the Western blot results. *n* = 4 per group for the Western blot experiment. *n* = 8 per group for the memory test. Note: Western blot gel images of subpanels B and C were obtained from the same basal forebrain tissues and the same GAPDH gel image was used.

Western blot showed that sleep deprivation led to increased BDNF expression in the basal forebrain, and ASO7 further enhanced the BDNF expression when combined with acute sleep deprivation (*F*_2,6_ = 20.72, *p* = .0020, Figure 3B). Six hours of sleep deprivation did not increase PSD95 protein expression, but ASO7 increased the PSD95 protein expression in sleep‐deprived mice (*F*_2,6_ = 7.058, *p* = .0265, Figure 3C). In the step‐down avoidance test, the latencies measured at 1 and 24 h after training were significantly increased in sleep deprivation plus ASO7 microinjection group (*F*_2,21_ = 6.011, *p* = .009, Figure 3D; *F*_2,21_ = 7.720, *p* < .003, Figure 3E, respectively) compared with that of sleep deprivation.

## 4 DISCUSSION

This study revealed that downregulation of ATXN2 mRNA and protein by ASO7 in basal forebrain increased BDNF expression in the basal forebrain and regulated BDNF and PSD95 expression in the hippocampus, which in turn enhanced spatial memory and partly reversed sleep deprivation‐induced fear memory impairments.

Sleep deprivation and chronic sleep restriction increase homeostatic sleep drive and impair waking neurobehavioral functions as reflected in attention, cognitive speed and memory (Goel et al., 2013). Basal forebrain is a critical region regulating sleep and wakefulness (Peng et al., 2020). Intertissue networks between the basal forebrain, hippocampus, and prefrontal cortex in rodent animals could be affected by disturbed sleep (Lagus et al., 2012). The hippocampus serves a critical function in memory and receives input from the medial septum of the basal forebrain cholinergic system. It is not surprising that the potential role of the septo‐hippocampal pathway has been proven to play a role in learning and memory (Kesner, 1988; Khakpai et al., 2013). We selected the basal forebrain as the brain area for drug injection in this study to observe whether basal forebrain BDNF signaling activation by ASO targeting ATXN2 mRNA could reverse the memory impairments induced by sleep deprivation.

We previously found that 6 h of sleep deprivation induced fear memory impairments and microinjection of BDNF into the basal forebrain mitigated the fear memory impairments caused by sleep deprivation (Ma et al., 2020). The results indicate that increasing BDNF expression in the basal forebrain is important in combating sleep deprivation‐induced cognitive impairments. The pathogenic mechanism of spinocerebellar ataxia associates with reduction in BDNF mRNA expression and abnormal localization of BDNF protein (Takahashi et al., 2012). Antisense oligonucleotide drugs (ASOs) directed at the ATXN2 gene had been administered to the central nervous system of SCA2 mouse models and had a dramatic effect on survival and improved motor function of mouse with SCA2 phenotypes (Becker et al., 2017; Scoles et al., 2017). More importantly, ASO7 could reduce cerebellar ATXN2 expression by 75% for more than 10 weeks (Scoles et al., 2017). So, we propose the hypothesis that inhibition of ATAXIN by ASO7 may consistently increase BDNF expression, thereby ameliorating memory impairments in sleep‐deprived mice.

Morris water maze (MWM) are classical tasks widely used to assess spatial learning and memory in rodents (Lissner et al., 2021; Liu et al., 2020). In this study, we used the MWM to see whether ASO7 microinjected into the basal forebrain could improve the spatial memory ability of normal mice and observed BDNF expression in basal forebrain and hippocampus (Figure 1A). The results suggested ASOs targeting ATXN2 could enhance spatial learning of mice (Figure 1D and E) and the inhibitory effect on ATXN2 mRNA and protein expression last more than one month (Figure 1F and G). The Western blot results showed BDNF protein expression in the basal forebrain and hippocampus was elevated (Figure 2A, B, E, and F). Our MWM results were consistent with previous research revealing that BDNF is an important regulator of synaptic transmission and long‐term potentiation (LTP) in the hippocampus and increases in hippocampal BDNF levels were associated with improved spatial learning and memory retention (El Hayek et al., 2019; Leal et al., 2014).

Acetylcholine has consistently been shown to be elevated in the hippocampus during various memory tasks, which demonstrated that cholinergic signaling from the medial septal nucleus and diagonal band nuclei in basal forebrain to the hippocampus is important for formation of spatial memories (Ballinger et al., 2016). Moreover, it was demonstrated that BDNF can stimulate the choline acetyltransferase activity in the basal forebrain (Liebl & Koo, 1994). Collectively, the present data indicated that ASOs targeting ATXN2 may activate cholinergic signaling in the basal forebrain by increasing BDNF expression and thus regulate BDNF protein expression in the hippocampus. However, whether the levels of choline acetyltransferase activity in the basal forebrain were increased by ASO7 targeting ATXN2 needs further confirmation.

The postsynaptic density protein‐95 (PSD95) played an important role in synaptic plasticity and memory formation (Coley & Gao, 2018; Ding et al., 2020; Jeong et al., 2019). Changes in dendritic spine density or structural reorganization of spines are also important for synaptic function and memory (Frank et al., 2018; Ultanir et al., 2007). The study found that inhibition of ATAXIN2 in the basal forebrain of mice increased the expression of PSD95 protein (Figure 2B, C, F, and G) and spine density (Figure 2H–J) in the hippocampus. Studies have reported that blockade of BDNF signaling could impair hippocampal PSD95 expression (Mizuno et al., 2003; Yang et al., 2014). Whether inhibition of ataxin‐2 increases PSD‐95 expression through BDNF signaling needs further investigation.

The relation between BDNF and ATXN gene is not clear. Some studies found that expression of BDNF is decreased in ATXN‐mutant mice and delivery of BDNF could ameliorate ATXN‐mutation‐related neurological diseases (Mellesmoen et al., 2018; Sheeler et al., 2021). In the present study, we found for the first time that inhibition of the basal forebrain ATXN2 gene increased the expression of BDNF in the basal forebrain (Figure 2A). TAR DNA binding protein 43 (TDP‐43) is a versatile RNA/DNA binding protein involved in RNA metabolism (Prasad et al., 2019). It was reported that abnormal TDP‐43 function could impair activity‐dependent BDNF secretion, synaptic plasticity, and cognitive behaviors and the decrease in ataxin‐2 protein could reduce aggregation of TDP‐43 (Becker et al., 2017; Tann et al., 2019). Therefore, there is a possibility that the increase of BDNF expression may be related to the suppressed TDP‐43 expression caused by reduced ataxin‐2 expression.

We demonstrated that inhibition of ATXN2 by ASOs improves memory in mice by increasing BDNF and PSD95 expression in the basal forebrain and hippocampus. Based on the findings above, we next applied ASOs to sleep‐deprived mice to observe whether it could ameliorate sleep deprivation‐induced cognitive impairments (Figure 3A). Previous studies have confirmed that sleep deprivation can cause fear memory impairments and activating BDNF/tyrosine receptor kinase (TrkB) signaling could improve memory deficits in rodents under sleep deprivation (Ma et al., 2020; Montes‐Rodríguez et al., 2019; Pace‐Schott et al., 2015; Pei et al., 2021). In this study, we found 6 h of sleep deprivation increased BDNF expression, which was consistent with previous research showing that acute sleep deprivation induced a fast increase in BDNF serum levels within hours (Giacobbo et al., 2016; Schmitt et al., 2016). Different sleep deprivation protocols caused different changes in the expression of PSD95. Studies showed that 4 h of sleep deprivation for 3 days or 96‐h paradoxical sleep deprivation did not alter the PSD‐95 expression in rats (Fernandes et al., 2013; Lopez et al., 2008), while 18‐h sleep deprivation for 3 days decreased PSD‐95 expression (Li et al., 2019). In this study, we did not find significant changes induced by 6‐h sleep deprivation but found increased BDNF and PSD95 in the basal forebrain of ASOs‐injected mice.

## 5 CONCLUSION

In summary, we successfully used a novel method, ASOs targeting ATXN2, to attenuate sleep deprivation‐induced fear memory impairments by modulation of BDNF signaling in the basal forebrain and hippocampus.

## AUTHOR CONTRIBUTIONS

**Hao Zhang and Wei Wang** designed the research, prepared the manuscript, and made the revision. **Tao Ma and Long Feng** participated in the whole set of experiments and wrote the manuscript. **Shi‐Nan Wei, Ying‐Xin Zhang, and Yang Chu** conducted the behavioral tests. **Wei Wang, Guan‐Hua Li, and Yan Lu** conducted the Western blot experiments. Yang Chu checked the data and made the statistical analysis. **Tao Ma and Long Feng** contributed equally to this work.

## CONFLICT OF INTEREST STATEMENT

The authors have no conflict of interest to declare.

### PEER REVIEW

The peer review history for this article is available at https://publons.com/publon/10.1002/brb3.3013.

## Data Availability

The data used during the study are available from the corresponding authors by request.

## References

[brb33013-bib-0001] Ballinger, E. C. , Ananth, M. , Talmage, D. A. , & Role, L. W. (2016). Basal forebrain cholinergic circuits and signaling in cognition and cognitive decline. Neuron, 91(6), 1199–1218. 10.1016/j.neuron.2016.09.006 27657448 PMC5036520

[brb33013-bib-0002] Bawari, S. , Tewari, D. , Argüelles, S. , Sah, A. N. , Nabavi, S. F. , Xu, S. , & Shirooie, S. (2019). Targeting BDNF signaling by natural products: Novel synaptic repair therapeutics for neurodegeneration and behavior disorders. Pharmacological Research, 148, 104458. 10.1016/j.phrs.2019.104458 31546015

[brb33013-bib-0003] Becker, L. A. , Huang, B. , Bieri, G. , Ma, R. , Knowles, D. A. , Jafar‐Nejad, P. , & Gitler, A. D. (2017). Therapeutic reduction of ataxin‐2 extends lifespan and reduces pathology in TDP‐43 mice. Nature, 544(7650), 367–371. 10.1038/nature22038 28405022 PMC5642042

[brb33013-bib-0005] Campos‐Jurado, Y. , Igual‐Lopez, M. , Padilla, F. , Zornoza, T. , Granero, L. , Polache, A. , & Hipolito, L. (2019). Activation of MORs in the VTA induces changes on cFos expression in different projecting regions: Effect of inflammatory pain. Neurochemistry International, 131, 104521. 10.1016/j.neuint.2019.104521 31419453

[brb33013-bib-0007] Coley, A. A. , & Gao, W. J. (2018). PSD95: A synaptic protein implicated in schizophrenia or autism? Progress in Neuro‐Psychopharmacology & Biological Psychiatry, 82, 187–194. 10.1016/j.pnpbp.2017.11.016 29169997 PMC5801047

[brb33013-bib-0008] Cousins, J. N. , & Fernández, G. (2019). The impact of sleep deprivation on declarative memory. Progress in Brain Research, 246, 27–53. 10.1016/bs.pbr.2019.01.007 31072562

[brb33013-bib-0010] Ding, B. , Lin, C. , Liu, Q. , He, Y. , Ruganzu, J. B. , Jin, H. , & Yang, W. (2020). Tanshinone IIA attenuates neuroinflammation via inhibiting RAGE/NF‐κB signaling pathway in vivo and in vitro. Journal of Neuroinflammation, 17(1), 302. 10.1186/s12974-020-01981-4 33054814 PMC7559789

[brb33013-bib-0011] Ding, M. , & Toth, L. A. (2006). mRNA expression in mouse hypothalamus and basal forebrain during influenza infection: A novel model for sleep regulation. Physiological Genomics, 24(3), 225–234. 10.1152/physiolgenomics.00005.2005 16403846

[brb33013-bib-0012] El Hayek, L. , Khalifeh, M. , Zibara, V. , Abi Assaad, R. , Emmanuel, N. , Karnib, N. , & Sleiman, S. F. (2019). Lactate mediates the effects of exercise on learning and memory through SIRT1‐dependent activation of hippocampal brain‐derived neurotrophic factor (BDNF). Journal of Neuroscience, 39(13), 2369–2382. 10.1523/jneurosci.1661-18.2019 30692222 PMC6435829

[brb33013-bib-0013] Fernandes, J. , Baliego, L. G. , Peixinho‐Pena, L. F. , de Almeida, A. A. , Venancio, D. P. , Scorza, F. A. , & Arida, R. M. (2013). Aerobic exercise attenuates inhibitory avoidance memory deficit induced by paradoxical sleep deprivation in rats. Brain Research, 1529, 66–73. 10.1016/j.brainres.2013.07.019 23895766

[brb33013-bib-0014] Frank, A. C. , Huang, S. , Zhou, M. , Gdalyahu, A. , Kastellakis, G. , Silva, T. K. , Lu, E. , Wen, X. , Poirazi, P. , Trachtenberg, J. T. , & Silva, A. J. (2018). Hotspots of dendritic spine turnover facilitate clustered spine addition and learning and memory. Nature Communication, 9(1), 422. 10.1038/s41467-017-02751-2 PMC5789055 29379017

[brb33013-bib-0015] Giacobbo, B. L. , Corrêa, M. S. , Vedovelli, K. , de Souza, C. E. , Spitza, L. M. , Gonçalves, L. , & Bromberg, E. (2016). Could BDNF be involved in compensatory mechanisms to maintain cognitive performance despite acute sleep deprivation? An exploratory study. International Journal of Psychophysiology, 99, 96–102. 10.1016/j.ijpsycho.2015.11.008 26602839

[brb33013-bib-0016] Gigante, A. F. , Lelli, G. , Romano, R. , Pellicciari, R. , Di Candia, A. , Mancino, P. V. , & Defazio, G. (2020). The relationships between ataxia and cognition in spinocerebellar ataxia type 2. Cerebellum (London, England), 19(1), 40–47. 10.1007/s12311-019-01079-5 31637587

[brb33013-bib-0017] Goel, N. , Basner, M. , Rao, H. , & Dinges, D. F. (2013). Circadian rhythms, sleep deprivation, and human performance. Progress in Molecular Biology and Translational Science, 119, 155–190. 10.1016/b978-0-12-396971-2.00007-5 23899598 PMC3963479

[brb33013-bib-0018] Hourez, R. , Servais, L. , Orduz, D. , Gall, D. , Millard, I. , de Kerchove d'Exaerde, A. , & Schiffmann, S. N. (2011). Aminopyridines correct early dysfunction and delay neurodegeneration in a mouse model of spinocerebellar ataxia type 1. Journal of Neuroscience, 31(33), 11795–11807. 10.1523/jneurosci.0905-11.2011 21849540 PMC6623197

[brb33013-bib-0019] Jeong, J. , Pandey, S. , Li, Y. , Badger, J. D. , 2nd, Lu, W. , & Roche, K. W. (2019). PSD‐95 binding dynamically regulates NLGN1 trafficking and function. PNAS, 116(24), 12035–12044. 10.1073/pnas.1821775116 31138690 PMC6575593

[brb33013-bib-0020] Kesner, R. P. (1988). Reevaluation of the contribution of the basal forebrain cholinergic system to memory. Neurobiology of Aging, 9(5‐6), 609–616. 10.1016/s0197-4580(88)80122-2 3062470

[brb33013-bib-0021] Khakpai, F. , Nasehi, M. , Haeri‐Rohani, A. , Eidi, A. , & Zarrindast, M. R. (2013). Septo‐hippocampo‐septal loop and memory formation. Basic and Clinical Neuroscience, 4(1), 5–23. 25337323 PMC4202558

[brb33013-bib-0022] Krause, A. J. , Simon, E. B. , Mander, B. A. , Greer, S. M. , Saletin, J. M. , Goldstein‐Piekarski, A. N. , & Walker, M. P. (2017). The sleep‐deprived human brain. Nature Reviews Neuroscience, 18(7), 404–418. 10.1038/nrn.2017.55 28515433 PMC6143346

[brb33013-bib-0023] Laffita‐Mesa, J. M. , Paucar, M. , & Svenningsson, P. (2021). Ataxin‐2 gene: A powerful modulator of neurological disorders. Current Opinion in Neurology, 34(4), 578–588. 10.1097/wco.0000000000000959 34010218 PMC8279897

[brb33013-bib-0024] Lagus, M. , Gass, N. , Saharinen, J. , Savelyev, S. , Porkka‐Heiskanen, T. , & Paunio, T. (2012). Inter‐tissue networks between the basal forebrain, hippocampus, and prefrontal cortex in a model for depression caused by disturbed sleep. Journal of Neurogenetics, 26(3‐4), 397–412. 10.3109/01677063.2012.694932 22783900

[brb33013-bib-0025] Leal, G. , Comprido, D. , & Duarte, C. B. (2014). BDNF‐induced local protein synthesis and synaptic plasticity. Neuropharmacology, 76(Pt C), 639–656. 10.1016/j.neuropharm.2013.04.005 23602987

[brb33013-bib-0026] Li, H. , Yu, F. , Sun, X. , Xu, L. , Miu, J. , & Xiao, P. (2019). Dihydromyricetin ameliorates memory impairment induced by acute sleep deprivation. European Journal of Pharmacology, 853, 220–228. 10.1016/j.ejphar.2019.03.014 30876981

[brb33013-bib-0027] Liebl, D. J. , & Koo, P. H. (1994). Monoamine‐activated alpha 2‐macroglobulin inhibits choline acetyltransferase of embryonic basal forebrain neurons and reversal of the inhibition by NGF and BDNF but not NT‐3. Journal of Neuroscience Research, 38(4), 407–414. 10.1002/jnr.490380406 7523691

[brb33013-bib-0028] Lissner, L. J. , Wartchow, K. M. , Toniazzo, A. P. , Gonçalves, C. A. , & Rodrigues, L. (2021). Object recognition and Morris water maze to detect cognitive impairment from mild hippocampal damage in rats: A reflection based on the literature and experience. Pharmacology Biochemistry and Behavior, 210, 173273. 10.1016/j.pbb.2021.173273 34536480

[brb33013-bib-0029] Liu, B. , Kou, J. , Li, F. , Huo, D. , Xu, J. , Zhou, X. , & Han, D. (2020). Lemon essential oil ameliorates age‐associated cognitive dysfunction via modulating hippocampal synaptic density and inhibiting acetylcholinesterase. Aging (Albany NY), 12(9), 8622–8639. 10.18632/aging.103179 32392535 PMC7244039

[brb33013-bib-0030] Looti Bashiyan, M. , Nasehi, M. , Vaseghi, S. , & Khalifeh, S. (2021). Investigating the effect of crocin on memory deficits induced by total sleep deprivation (TSD) with respect to the BDNF, TrkB and ERK levels in the hippocampus of male Wistar rats. Journal of Psychopharmacology, 35(6), 744–754. 10.1177/02698811211000762 33899577

[brb33013-bib-0031] Lopez, J. , Roffwarg, H. P. , Dreher, A. , Bissette, G. , Karolewicz, B. , & Shaffery, J. P. (2008). Rapid eye movement sleep deprivation decreases long‐term potentiation stability and affects some glutamatergic signaling proteins during hippocampal development. Neuroscience, 153(1), 44–53. 10.1016/j.neuroscience.2008.01.072 18359575 PMC2389877

[brb33013-bib-0032] Ma, T. , Zhang, H. , Xu, Z. P. , Lu, Y. , Fu, Q. , Wang, W. , & Mi, W. D. (2020). Activation of brain‐derived neurotrophic factor signaling in the basal forebrain reverses acute sleep deprivation‐induced fear memory impairments. Brain and Behavior, 10(4), e01592. 10.1002/brb3.1592 32157827 PMC7177564

[brb33013-bib-0033] Mellesmoen, A. , Sheeler, C. , Ferro, A. , Rainwater, O. , & Cvetanovic, M. (2018). Brain derived neurotrophic factor (BDNF) delays onset of pathogenesis in transgenic mouse model of spinocerebellar ataxia type 1 (SCA1). Frontiers in Cellular Neuroscience, 12, 509. 10.3389/fncel.2018.00509 30718999 PMC6348256

[brb33013-bib-0034] Mizuno, M. , Yamada, K. , He, J. , Nakajima, A. , & Nabeshima, T. (2003). Involvement of BDNF receptor TrkB in spatial memory formation. Learning & Memory (Cold Spring Harbor, N.Y.), 10(2), 108–115. 10.1101/lm.56003 PMC196664 12663749

[brb33013-bib-0035] Montes‐Rodríguez, C. J. , Rueda‐Orozco, P. E. , & Prospéro‐García, O. (2019). Total sleep deprivation impairs fear memory retrieval by decreasing the basolateral amygdala activity. Brain Research, 1719, 17–23. 10.1016/j.brainres.2019.05.030 31128099

[brb33013-bib-0036] Nagahara, A. H. , & Tuszynski, M. H. (2011). Potential therapeutic uses of BDNF in neurological and psychiatric disorders. Nature Reviews Drug Discovery, 10(3), 209–219. 10.1038/nrd3366 21358740

[brb33013-bib-0037] Pace‐Schott, E. F. , Germain, A. , & Milad, M. R. (2015). Effects of sleep on memory for conditioned fear and fear extinction. Psychological Bulletin, 141(4), 835–857. 10.1037/bul0000014 25894546 PMC4486610

[brb33013-bib-0038] Pei, W. , Meng, F. , Deng, Q. , Zhang, B. , Gu, Y. , Jiao, B. , & Ding, Y. (2021). Electroacupuncture promotes the survival and synaptic plasticity of hippocampal neurons and improvement of sleep deprivation‐induced spatial memory impairment. CNS Neuroscience & Therapeutics, 27(12), 1472–1482. 10.1111/cns.13722 34623740 PMC8611786

[brb33013-bib-0039] Peng, W. , Wu, Z. , Song, K. , Zhang, S. , Li, Y. , & Xu, M. (2020). Regulation of sleep homeostasis mediator adenosine by basal forebrain glutamatergic neurons. Science, 369(6508). 10.1126/science.abb0556 32883833

[brb33013-bib-0040] Prasad, A. , Bharathi, V. , Sivalingam, V. , Girdhar, A. , & Patel, B. K. (2019). Molecular mechanisms of TDP‐43 misfolding and pathology in amyotrophic lateral sclerosis. Frontiers in Molecular Neuroscience, 12, 25. 10.3389/fnmol.2019.00025 30837838 PMC6382748

[brb33013-bib-0041] Rahmani, M. , Rahmani, F. , & Rezaei, N. (2020). The brain‐derived neurotrophic factor: missing link between sleep deprivation, insomnia, and depression. Neurochemical Research, 45(2), 221–231. 10.1007/s11064-019-02914-1 31782101

[brb33013-bib-0042] Schmitt, K. , Holsboer‐Trachsler, E. , & Eckert, A. (2016). BDNF in sleep, insomnia, and sleep deprivation. Annals of Medicine, 48(1‐2), 42–51. 10.3109/07853890.2015.1131327 26758201

[brb33013-bib-0043] Scoles, D. R. , Meera, P. , Schneider, M. D. , Paul, S. , Dansithong, W. , Figueroa, K. P. , & Pulst, S. M. (2017). Antisense oligonucleotide therapy for spinocerebellar ataxia type 2. Nature, 544(7650), 362–366. 10.1038/nature22044 28405024 PMC6625650

[brb33013-bib-0044] Sheeler, C. , Rosa, J. G. , Borgenheimer, E. , Mellesmoen, A. , Rainwater, O. , & Cvetanovic, M. (2021). Post‐symptomatic delivery of brain‐derived neurotrophic factor (BDNF) ameliorates spinocerebellar ataxia type 1 (SCA1) pathogenesis. Cerebellum (London, England), 20(3), 420–429. 10.1007/s12311-020-01226-3 33394333 PMC8217121

[brb33013-bib-0045] Takahashi, M. , Ishikawa, K. , Sato, N. , Obayashi, M. , Niimi, Y. , Ishiguro, T. , & Mizusawa, H. (2012). Reduced brain‐derived neurotrophic factor (BDNF) mRNA expression and presence of BDNF‐immunoreactive granules in the spinocerebellar ataxia type 6 (SCA6) cerebellum. Neuropathology, 32(6), 595–603. 10.1111/j.1440-1789.2012.01302.x 22393909

[brb33013-bib-0046] Tann, J. Y. , Wong, L. W. , Sajikumar, S. , & Ibáñez, C. F. (2019). Abnormal TDP‐43 function impairs activity‐dependent BDNF secretion, synaptic plasticity, and cognitive behavior through altered Sortilin splicing. Embo Journal, 38(5). 10.15252/embj.2018100989 PMC6396160 30692134

[brb33013-bib-0047] Ultanir, S. K. , Kim, J. E. , Hall, B. J. , Deerinck, T. , Ellisman, M. , & Ghosh, A. (2007). Regulation of spine morphology and spine density by NMDA receptor signaling in vivo. PNAS, 104(49), 19553–19558. 10.1073/pnas.0704031104 18048342 PMC2148327

[brb33013-bib-0049] Wang, F. , Wei, X. X. , Chang, L. S. , Dong, L. , Wang, Y. L. , & Li, N. N. (2021). Ultrasound combined with microbubbles loading BDNF retrovirus to open blood brain barrier for treatment of Alzheimer's disease. Frontiers in Pharmacology, 12, 615104. 10.3389/fphar.2021.615104 33746754 PMC7973107

[brb33013-bib-0050] Xu, M. , Chung, S. , Zhang, S. , Zhong, P. , Ma, C. , Chang, W.‐C. , & Dan, Y. (2015). Basal forebrain circuit for sleep‐wake control. Nature Neuroscience, 18(11), 1641–1647. 10.1038/nn.4143 26457552 PMC5776144

[brb33013-bib-0051] Yang, J. L. , Lin, Y. T. , Chuang, P. C. , Bohr, V. A. , & Mattson, M. P. (2014). BDNF and exercise enhance neuronal DNA repair by stimulating CREB‐mediated production of apurinic/apyrimidinic endonuclease 1. NeuroMolecular Medicine, 16(1), 161–174. 10.1007/s12017-013-8270-x 24114393 PMC3948322

[brb33013-bib-0053] Zhuang, Y. , Guan, Y. , Qiu, L. , Lai, M. , Tan, M. T. , & Chen, P. (2018). A novel rank‐based non‐parametric method for longitudinal ordinal data. Statistical Methods in Medical Research, 27(9), 2775–2794. 10.1177/0962280216686628 28067124

